# Long exposure to mature ooplasm can alter DNA methylation at imprinted loci in non-growing oocytes but not in prospermatogonia

**DOI:** 10.1530/REP-13-0359

**Published:** 2014-01

**Authors:** Yayoi Obata, Takuya Wakai, Satoshi Hara, Tomohiro Kono

**Affiliations:** Department of BioscienceTokyo University of Agriculture1-1-1 Sakuragaoka, Setagaya-ku, Tokyo, 156-8502Japan

## Abstract

DNA methylation imprints that are established in spermatogenesis and oogenesis are essential for functional gametes. However, the mechanisms underlying gamete-specific imprinting remain unclear. In this study, we investigated whether male and female gametes derived from newborn mice are epigenetically plastic and whether DNA methylation imprints are influenced by the niche surrounding the nuclei of the gametes. When prospermatogonia possessing sperm-specific DNA methylation imprints were fused with enucleated fully grown oocytes and exposed to the ooplasm for 5–6 days, the DNA methylation status of the reconstituted oocytes remained identical to that of prospermatogonia for all the imprinted regions analysed. These results suggest that the imprinting status of prospermatogonia is stable and that the epigenome of prospermatogonia loses sexual plasticity. By contrast, when non-growing oocytes lacking oocyte-specific DNA methylation imprints were fused with enucleated fully grown oocytes and the reconstituted oocytes were then cultured for 5–6 days, the *Igf2r*, *Kcnq1ot1* and, unexpectedly, *H19/Igf2* differentially methylated regions (DMRs) were methylated. Methylation imprints were entirely absent in oocytes derived from 5-day-old mice, and *H19/Igf2* DMR is usually methylated only in spermatogenesis. These findings indicate that in the nuclei of non-growing oocytes the chromatin conformation changes and becomes permissive to DNA methyltransferases in some DMRs and that mechanisms for maintaining non-methylated status at the *H19/Igf2* DMR are lost upon long exposure to mature ooplasm.

## Introduction

DNA methylation imprints that are established in spermatogenesis and oogenesis are maintained after fertilisation, which results in parental-origin-specific gene expression in the somatic cell lineage. By contrast, in the germ cell lineage, parental-origin-specific DNA methylation imprints are erased and gametes acquire new imprints according to their own sex (Ferguson-Smith 2011, Obata 2011).

It has been reported that the maintenance of allele-specific DNA methylation is required for protection against DNA demethylation by pluripotency-associated protein 3 (DPPA3; Nakamura *et al*. 2007, 2012), maintenance of DNA methylation by DNA methyltransferase 1 (DNMT1; Li *et al*. 1993), and maintenance of the unmethylated status of differentially methylated regions (DMRs) by CTCF (Schoenherr *et al*. 2003, Szabo *et al*. 2004). During the erasure of DNA methylation imprints in the germ cell lineage, maintenance mechanisms for DNA methylation imprints must be disrupted, although the precise nature of such disruption remains unknown.

DNMT3A and DNMT3L are essential for the acquisition of DNA methylation imprints in both male and female gametes (Bourc'his *et al*. 2001, Hata *et al*. 2002, Kaneda *et al*. 2004). Sperm-specific DNA methylation imprints are established during the mitotic arrest of prospermatogonia in foetal testes (Davis *et al*. 2000, Hiura *et al*. 2007), whereas oocyte-specific DNA methylation imprints are established during oocyte growth, and oocytes possessing all imprints first appear in the peri-pubertal stage (Lucifero *et al*. 2004, Hiura *et al*. 2006).

We have demonstrated that oocytes obtained from sex-reversed XY female B6.Y^TIR^ mice do not naturally develop beyond the four-cell stage (Villemure *et al*. 2007), but can develop to term after cytoplasmic exchange with oocytes from XX females, IVF and embryo transfer (Obata *et al*. 2008). Therefore, the nuclei of oocytes from XY females are able to acquire oocyte-specific imprints. However, unless sex reversal occurs, B6.Y^TIR^ mice are fully fertile males. Thus, germ cells are epigenetically bipotential in the early stage and can acquire either sperm- or oocyte-specific imprints independent of their genetic sex. Sex reversal in B6.Y^TIR^ mice is caused by the delayed and decreased expression of *Sry* in gonadal somatic cells (Park *et al*. 2011), which suggests that the niche surrounding germ cells controls the sex of the gametes, including their imprinting.

In this study, to investigate whether epigenetic plasticity is maintained in male and female gametes derived from newborn mice and whether DNA methylation imprints are influenced by the niche surrounding the nuclei of gametes, prospermatogonia possessing sperm-specific DNA methylation imprints and non-growing oocytes lacking oocyte-specific DNA methylation imprints were fused with fully grown oocytes respectively. The reconstituted oocytes were cultured for 5–6 days and then subjected to DNA methylation analysis of imprinted genes.

## Materials and methods

### Animals

All the procedures used in this study were reviewed and approved by the Tokyo University of Agriculture Institutional Animal Care and Use Committee and were carried out in accordance with the Guidelines for Proper Conduct of Animal Experiments, as established by the Science Council of Japan. BDF1 (C57BL/6N×DBA/2; CLEA Japan, Tokyo, Japan) hybrid mice were used for all the experiments. JF1 mice (*Mus musculus molossinus*) were used only in the polymorphic analysis of DNA methylation (Koide *et al*. 1998).

### Prospermatogonia and oocytes

Prospermatogonia and non-growing oocytes were collected from newborn mice (0–2 days *postpartum*). Testes and ovaries were treated with 0.1% collagenase (Wako, Osaka, Japan) in BSA-free M2 medium for 20 min at 37 °C and were then treated with 0.05% trypsin–0.53 mM EDTA (Gibco, Life Technologies Japan) in PBS for 15 min at 37 °C. The cells of the testes and ovaries were then dispersed and suspended by pipetting in M2 medium containing 10 μg/ml cytochalasin B (Sigma–Aldrich Japan), 100 ng/ml demecolcine (Wako) and 240 μM dibutyryl cAMP (Sigma–Aldrich Japan).

Cumulus–oocyte complexes (COCs) were collected from the antral follicle of the ovaries of adult female mice 40–42 h after the injection of 5 IU of equine chorionic gonadotrophin (Serotropin; ASKA Pharmaceutical Company, Tokyo, Japan). Fully grown oocytes at the germinal vesicle (GV) stage were denuded by pipetting. Cumulus cells were used for the reconstruction of COCs.

### Fusion of ooplasm and gametes

**Figure 1:**
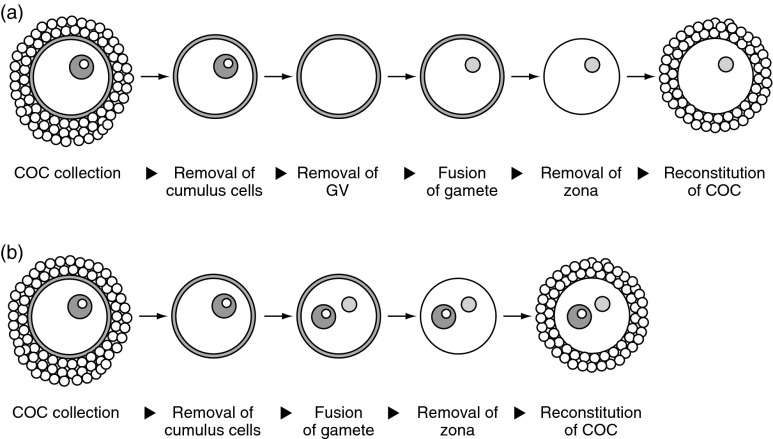
Schematic diagram of COC reconstitution. (a) GV is removed from a fully grown oocyte before fusion. The prospermatogonium or non-growing oocyte is fused with the enucleated oocyte. (b) GV is not removed from a fully grown oocyte before fusion. The prospermatogonium or non-growing oocyte derived from a JF1 newborn mouse is fused with the intact GV oocyte from a BDF1 adult mouse for polymorphic analysis of DNA methylation.

Fusion experiments were conducted in M2 medium containing 10 μg/ml cytochalasin B, 100 ng/ml demecolcine and 240 μM dibutyryl cAMP using a nuclear transfer technique. Prospermatogonia and non-growing oocytes were fused with enucleated or intact fully grown oocytes respectively using an inactivated Sendai virus (2700 haemagglutinating activity (units/ml)). As a control, the GVs of fully grown oocytes were fused with enucleated fully grown oocytes. After fusion, the zona pellucida was removed mechanically using a micromanipulator with a glass needle. To reconstitute COCs, these oocytes were co-cultured with cumulus cells in α-MEM (Gibco, 12000-022; Life Technologies Japan) supplemented with 2.2 g/l NaHCO_3_, 3.5 g/l glucose, 240 μM dibutyryl cAMP and 5% FBS in 5% CO_2_, 5% O_2_ and 90% N_2_ at 37 °C for 5 days. Approximately 25 oocytes were co-cultured with cumulus cells isolated from 60 COCs in a 100 μl drop of medium covered with paraffin oil, and half of the medium was exchanged every 2 days (Fig. 1).

### DNA methylation analysis

Prior to lysis, reconstituted COCs were treated with 0.05% trypsin–0.53 mM EDTA in PBS, and cumulus cells were completely removed by pipetting. Approximately 200 intact gametes or reconstituted oocytes were pooled in 18 μl of lysis buffer (1% SDS, 5 μg of proteinase K and 2 μg of yeast tRNA) and then incubated at 37 °C for 1 h to extract DNA. We used the CpGenome DNA Modification Kit (Millipore, Billerica, MA, USA) for sodium bisulphite conversion of DNA. DNA methylation was assessed at a DMR in each of the five imprinted regions: *Igf2r*, *Kcnq1ot1*, *Mest*, *H19/Igf2* and *Dlk1/Gtl2*. Primers and PCR conditions are summarised in Supplementary Table 2, see section on supplementary data given at the end of this article. Bisulphite-modified DNA from 40 to 80 gametes was subjected to PCR for each gene. PCR products were cloned into a T Easy vector (pGEM T Easy vector; Promega) and then sequenced using an Applied Biosystems sequencing system (ABI PRISM 3100, Tokyo, Japan). Three pools of DNAs were analysed independently. Differences in methylation levels were assessed using the Mann–Whitney *U* test.

### Immunostaining

For immunostaining of DNMT3A and DNMT3L (Sakai *et al*. 2004, Hirasawa *et al*. 2008), reconstituted oocytes were fixed and permeabilised with 2% paraformaldehyde and 0.2% Triton X-100 in PBS for 1 h at room temperature. Then, the oocytes were incubated in PBS supplemented with 3% BSA for 1 h at room temperature. After blocking, the oocytes were incubated with mouse anti-DNMT3L polyclonal antibody (gifted from Shoji Tajima; dilution rate 1:100) overnight at 4 °C followed by incubation with DNMT3A MAB (IMGENEX, San Diego, CA, USA; IMG-268A CA; dilution rate 1:300). The oocytes were washed with PBS containing 0.1% Triton X-100 and 1% BSA for 20 min each. Subsequently, the oocytes were incubated with Alexa488-conjugated anti-mouse IgG for DNMT3A detection (Molecular Probes; dilution rate 1:500) and Alexa568-conjugated anti-rabbit IgG for DNMT3L detection (Life Technologies, Grand Island, NY, USA; dilution rate 1:500). Nuclei were counterstained using DAPI. The oocytes were observed using a confocal laser scanning microscope (LSM710, Carl Zeiss Japan, Tokyo, Japan).

## Results and discussion

### Fusion of ooplasm and gametes

To determine whether DNA methylation imprints are influenced by the niche surrounding the nuclei of the gametes, we conducted fusion experiments using nuclear transfer technique and COC reconstitution for long exposure of nuclei derived from gametes in the cytoplasm of fully grown oocytes (Fig. 1).

Prior to the fusion experiments, cumulus cells should be removed from GV oocytes. However, denuded oocytes were not viable within 48 h. Therefore, denuded oocytes were co-cultured with cumulus cells after the removal of the zona. Cumulus cells adhered tightly to zona-free oocytes, and the resultant oocytes had a healthy appearance (Supplementary Figure 1, see section on supplementary data given at the end of this article and Supplementary Table 1). When 7 or more days had elapsed, some oocytes were denuded naturally. Hence, we assessed the DNA methylation status within 5–6 days of the initiation of culture.

### Imprinting status of nuclei derived from prospermatogonia after long exposure to ooplasm

**Figure 2:**
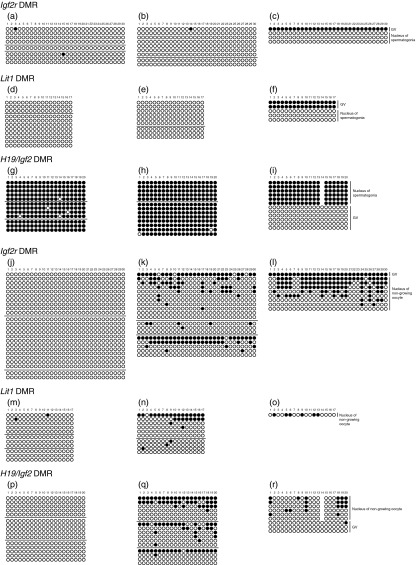
DNA methylation status in reconstituted oocytes after 5–6 days of culture. (a, d and g) DNA methylation status of prospermatogonia. (b, e and h) DNA methylation status of oocytes containing nuclei derived from prospermatogonia after 5–6 days of culture. (c, f and i) DNA methylation status of oocytes containing nuclei derived from prospermatogonia and GV after 5–6 days of culture. (j, m and p) DNA methylation status of non-growing oocytes. (k, n and q) DNA methylation status of oocytes containing nuclei derived from non-growing oocytes after 5–6 days of culture. (l, o and r) DNA methylation status of oocytes containing nuclei derived from non-growing and fully grown oocytes after 5–6 days of culture. Filled circle, methylated cytosine; open circle, unmethylated cytosine. A line of circles represents a single DNA strand. Each pool of oocytes is distinguishable by the horizontal lines.

As has been reported previously (Davis *et al*. 2000, Hiura *et al*. 2007), *H19/Igf2* and *Dlk1/Gtl2* intergenic (IG) DMRs were fully methylated, whereas *Igf2r*, *Kcnq1ot1* and *Mest* DMRs were not methylated in prospermatogonia derived from newborn mice (Fig. 2 and Table 1). Prospermatogonia were fused with enucleated or intact fully grown oocytes, and these oocytes were then cultured with cumulus cells for 5–6 days. However, the DNA methylation status of the reconstituted oocytes was not altered; it remained identical to that of prospermatogonia in all the analysed regions (Fig. 2 and Table 1). Therefore, it is likely that the imprinting status of prospermatogonia is stable and that the epigenome of prospermatogonia loses sexual plasticity. However, Wang *et al*. (2012) reported that spermatogonial cells from 8-day-old mice differentiated into oocyte-like cells and that, surprisingly, sperm-specific imprints were converted into oocyte-specific imprints over a culture period of 3 weeks. This appears to contradict our results; it is possible that spermatogonial stem cells cultured under certain conditions de-differentiated to primordial germ cell-like cells and the sperm-specific imprints were erased prior to development into oocyte-like cells.

### Imprinting status of nuclei derived from non-growing oocytes after long exposure to ooplasm

In non-growing oocytes derived from newborn mice, *H19/Igf2*, *Dlk1/Gtl2* IG, *Igf2r*, *Kcnq1ot1* and *Mest* DMRs were not methylated (Fig. 2 and Table 1). These non-growing oocytes were fused with enucleated or intact fully grown oocytes, and fused oocytes were then cultured with cumulus cells. After 5–6 days of culture, *Igf2r* and *Kcnq1ot1* DMRs showed 0–98.6% methylation in nuclei derived from non-growing oocytes. Furthermore, the *H19/Igf2* DMR, which is hypermethylated only in spermatogenesis, was also methylated (Fig. 2). *De novo* DNA methylation at the DMRs analysed did not occur in oocytes derived from 5-day-old mice. As a control experiment, nuclei derived from fully grown oocytes were fused with enucleated fully grown oocytes and these oocytes were cultured with cumulus cells for 5–6 days. However, the *H19/Igf2* DMR was not methylated (Table 1), which indicates that the DNA methylation of the *H19/Igf2* DMR in nuclei derived from non-growing oocytes was caused by long exposure to the mature ooplasm niche rather than due to micromanipulation. These results suggest that in the nuclei of non-growing oocytes mechanisms for maintaining the unmethylated status are lost or that the chromatin conformation changes and becomes permissive to DNMTs in some DMRs upon exposure to the mature ooplasm niche.

**Figure 3:**
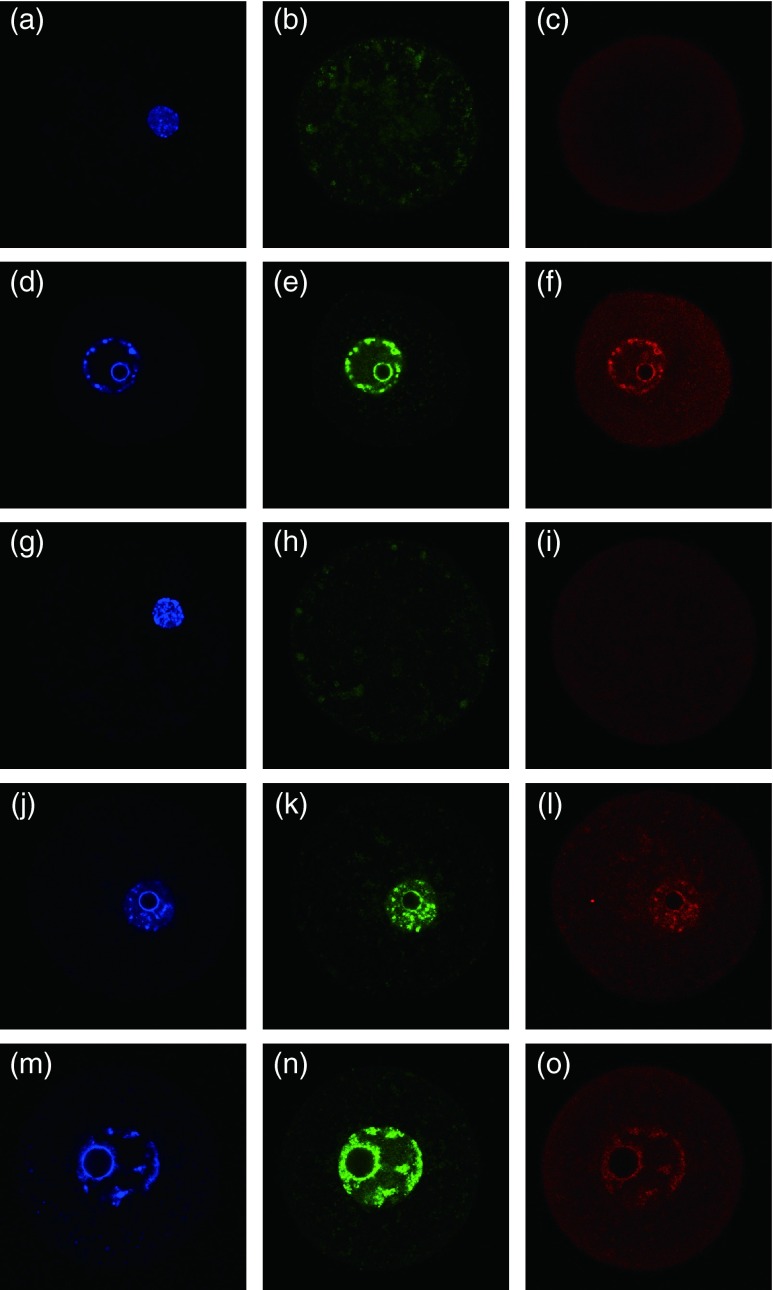
Immunostaining of reconstituted oocytes after 5 days of culture. (a, b and c) Oocyte containing nucleus derived from a prospermatogonium immediately after fusion. (d, e and f) Oocyte containing nucleus derived from a prospermatogonium after 5 days of culture. (g, h and i) Oocyte containing nucleus derived from a non-growing oocyte immediately after fusion. (j, k and l) Oocyte containing nucleus derived from a non-growing oocyte after 5 days of culture. (m, n and o) GV oocyte. Blue, DAPI (nucleus); green, DNMT3A and red, DNMT3L.

To confirm that this methylation was induced by DNMT3A and DNMT3L, we carried out immunostaining in the oocytes fused with gametes (Fig. 3). Immediately after fusion, nuclei derived from prospermatogonia and non-growing oocytes were condensed, and DNMT3A and DNMT3L were weakly detected in the nuclei of prospermatogonia but were absent in the nuclei of non-growing oocytes. Five days after the initiation of culture, the nuclei had swelled, and DNMT3A and DNMT3L were detected in both types of nuclear-transferred oocytes. Some nucleoli clearly appeared in the nucleus. It is not known whether DNMT3A and DNMT3L were translated from the mRNA of the recipient cytoplasm or from the newly synthesised mRNA of the donor nuclei in the present study. Regardless, the existence of DNMT3A and DNMT3L in the nuclei of non-growing oocytes must be related to the alteration in DNA methylation. By contrast, when nuclei derived from non-growing oocytes were exposed to the mature ooplasm niche, the DNA methylation status of *Mest* was not affected (Table 1). It is known that oocyte-specific DNA methylation imprints are established with gene-specific timing. *Mest* is a gene whose imprinting is established in the later stage of oocyte growth (Obata & Kono 2002, Lucifero *et al*. 2004, Hiura *et al*. 2006). Whether DNA methylation is induced in nuclei derived from non-growing oocytes by exposure to the mature ooplasm niche may depend on the mechanisms for the establishment of imprinting in each gene. Furthermore, in oocytes containing the nuclei of prospermatogonia, *Igf2r* and *Kcnq1ot1* DMRs did not enable epigenetic alteration in spite of nuclear swelling and DNMT3A and DNMT3L reactivation. This finding indicates that oocyte-specific methylated regions are shielded from DNMT3A and DNMT3L in prospermatogonia even after long exposure to the ooplasm niche.

In mice, sexual dimorphism of germ cells is obvious in the embryonal gonads at 13.5 days postcoitum (dpc). Male germ cells show arrested mitosis, whereas female germ cells enter meiosis (McLaren 1991). Prior to the sex differentiation of germ cells, *Sry* and *Sox9* are expressed in the somatic cells in the male gonad, which leads to testes cord formation and Sertoli and Leydig cell differentiation (Kashimada & Koopman 2010). Adams & McLaren (2002) demonstrated that the timing of entry into meiosis or arrest at mitosis in germ cells is governed by gonadal somatic cells until 12.5–13.5 dpc and that germ cells have bipotential independent of their genetic sex until 11.5 dpc. From our results, it is unclear whether the timing of the commitment to mitosis/meiosis is concomitant with the timing of commitment to sperm/oocyte-specific imprinting. However, it is intriguing that the *H19/Igf2* DMR was methylated in nuclei derived from non-growing oocytes that had already entered into meiosis. It is known that CTCF binds to specific sites in the non-methylated *H19/Igf2* DMR (Schoenherr *et al*. 2003, Szabo *et al*. 2004). *CTCF* knockdown during oogenesis induces the methylation of the *H19/Igf2* DMR in oocytes (Fedoriw *et al*. 2004). Thus, it is likely that the maintenance of the non-methylated status of DMRs is important for establishing sperm- or oocyte-specific methylation imprints.

## Supplementary data

This is linked to the online version of the paper at http://dx.doi.org/10.1530/REP-13-0359.

## Figures and Tables

**Table 1:** Rate of methylated CpG sites at each imprinted locus in reconstituted oocytes after 5–6 days of culture.

	**Prospermatogonia**		**Non-growing oocytes**			**GV oocytes**
**Donor nuclei**	Intact	Exposed	Intact	Intact^a^	Exposed	Exposed
*Igf2r*	0.8	0.3	0	0	27.7*	99.6
*Kcnq1ot1*	0	0	1.1	1.5	18.7*	97.1
*Mest*	0	0.4	0.7	0.7	1.4	100
*H19/Igf2*	97.2	99.5	0	0.8	23.0*	0.8
*Dlk1/Gtl2*	100	99.1	0.5	0.3	1.7	0.5

*P<0.05.

a Oocytes derived from 5-day-old mice.
